# Minimizing bugs in cognitive neuroscience programming

**DOI:** 10.3389/fpsyg.2014.01435

**Published:** 2014-12-17

**Authors:** Vadim Axelrod

**Affiliations:** ^1^The Gonda Multidisciplinary Brain Research Center, Bar Ilan UniversityRamat Gan, Israel; ^2^Institute of Cognitive Neuroscience, University College LondonLondon, UK

**Keywords:** programming, bugs, code quality, minimizing errors, code testing

## Introduction

Rigorous data analysis is a cornerstone of empirical scientific research. In recent years, much attention in the field of cognitive neuroscience has been paid to the issue of correct statistical procedures (e.g., Kriegeskorte et al., 2009; Nieuwenhuis et al., 2011; Kilner, 2013). However, an additional essential aspect of data analysis, which has attracted relatively little attention, is the errors (bugs) in custom data analysis programming code. Whereas, in its broad definition a bug can be any type of an error, here I refer to it as a problem in the code that *does not* lead to a failure (error or crash) during execution; that is, code that contains a bug completes its execution properly, but the output result is incorrect. Notably, if an erroneous output result consists of reasonable values, then the programmer might have no indication that something went wrong. It is impossible to estimate how many published studies contain results with bugs; however, given the ubiquity of bugs in non-scientific (industrial) software (Zhivich and Cunningham, 2009), it is plausible that academic code is no exception. Furthermore, the quality of custom code in cognitive neuroscience field in particular, is probably even worse than in the industry because (a) code in the field of cognitive neuroscience is usually written by people with only basic programming training—after all, they are brain researchers and not software engineers; (b) the code most often is programmed by a single researcher, without any code peer-review procedure (Wiegers, 2002); and (c) the custom code is usually used by a single or few lab members only. This last point is critical, because when the code is used by many, as is the case with large open-source projects like SPM (http://www.fil.ion.ucl.ac.uk/spm/), FieldTrip (http://fieldtrip.fcdonders.nl/), EEGLab (http://sccn.ucsd.edu/eeglab/) or PyMVPA (http://www.pymvpa.org/), the likelihood is low that a serious bug would remain unnoticed for a long time. The goal of this paper is to give practical advice to cognitive neuroscientists on how to minimize bugs in their custom data analysis code. The advice is illustrated using MATLAB schematic samples; executable code examples can be found in the Supplementary Materials.

## Advice 1: the dangers of irrelevant data

Consider a case of an fMRI data analysis, where a researcher needs to calculate an average level of activation (e.g., z-score) for the predefined regions of interest (ROIs). The code logic goes as follows: (1) iterate over ROI files, and get the coordinates for each ROI; (2) for each voxel in the ROI, retrieve its z-score value; and (3) average values across ROIs' voxels, and store all the results in a table. Please see the code sample 1 in Box 1.

Box 1 **Code Sample 1**


for roi_id=1: num_ROIs
          roi_coord_matrix_XYZ = GetROICoordinates(ROIs_file_names{roi_id});
          for voxel_id=1:size(roi_coord_matrix_XYZ,1)
                    roi_zscore_vector(voxel_id) = GetVoxelZscore(roi_coord_matrix_XYZ(voxel_id,:));
          end

          Activation_summary{roi_id,1} = ROIs_file_names{roi_id};
          Activation_summary{roi_id,2} = mean(roi_zscore_vector);
end

**Code Sample 2:**

for roi_id=1: num_ROIs
          roi_coord_matrix_XYZ = GetROICoordinates(ROIs_file_names{roi_id});
          Activation_summary{roi_id,1} = ROIs_file_names{roi_id};
          Activation_summary{roi_id,2} = mean(GetROIZscores(roi_coord_matrix_XYZ));
end

**Code Sample 3:**

function generated_rois = CreateRightHemisphereROIs(ROIs_names_vec)

          for roi_id=1:length(ROIs_names_vec)
                    generated_rois{roi_id} = CreateROI(ROIs_names_vec{roi_id})

                    if (generated_rois{roi_id}.X_coord_center < 0)
                              error('Oops…Negative ROI X center coordinate. We Stop.');
                    end
          end

The problem with this code is that the *roi_zscore_vector* variable is not cleaned up between ROI loop cycles. So, if, for example, ROI No. 3 consists of 127 voxels and ROI No. 4 consists of only 7, then the mean result for ROI No. 4 will be based on 7 correct z-score values and 120 incorrect z-score values from the previous loop cycle. The easy way to fix the bug is to clean up the *roi_zscore_vector* variable between loop cycles (e.g., *roi_zscore_vector=[];*). It worth noting that a more secure and efficient way to write the code would be to retrieve all ROI's z-score values at once, instead of iterating over the single voxels (“vectorized” approach). Please see the code sample 2 in Box 1.

Bugs caused by irrelevant data might be as simple as the use of an incorrect index in the loop, misuse of the variable or the use of incorrect configuration file. These bugs usually occur as a result of an inaccuracy or momentary attentional lapse. There is no complete remedy for this problem, but adopting good coding practices (see Advice 4: Bugs and Code Quality) decreases the likelihood of such errors.

## Advice 2: test your code!

A fact not immediately appreciated by inexperienced programmers is that regardless of how hard one tries, it is difficult *not* to make errors during programming. The important thing is to catch these errors in time, so they do not remain unnoticed (i.e., become bugs). To this extent, test procedures substantially improve code quality. Below are several recommendations:

### Use a debugger to validate whether your code works as expected

Once the code is written, debug it first before fully executing it from beginning to end. That is, set a breakpoint(s) within the critical parts of the code; then, by using debugger step functionality, validate line by line that the flow of the program is as expected and the intermediate calculations are correct. In general, no quality code can be produced without using a debugger.

### Keep track and validate intermediate results

Keep track of intermediate calculation results by outputting them to command window, and preferably, also by saving them in a log file(s) or as graphical figures. This approach allows you: (a) to establish whether something went wrong during execution, and (b) to compare intermediate results between code executions.

### Compare automatic results with those created manually

A useful way to validate whether the results of custom code are correct is to compare them with the results generated manually. For example, if your code automatically converts z-score fMRI t-contrast images to binary masks, it is worth comparing the automatically generated masks with the masks created manually (e.g., using SPM's imcalc).

### Use simulated (surrogate) data to test the code

The use of a simulated dataset as opposed to real data is the only unbiased way to ensure that the code works as expected and does not contain bugs. The simulated dataset can help to ensure that the code finds the effect of interest when the data indeed contains it, and also that no effect is found when the data is a random noise. PyMVPA provides an excellent example of surrogate data use.

## Advice 3: protective (preventive) programming

The essence of the protective programming approach is that the program validates its intermediate results as it runs, and alerts the programmer in real time in the event of a problem. Consider, for example, the code that creates a collection of ROI masks for the right hemisphere according to the Montreal Neurological Institute (MNI) coordinate system. Accordingly, the X coordinate of all ROIs should be positive. Please see the code sample 3 in Box 1.

If for some reason the center coordinate of the created ROI was negative, then the code would just stop. Critically, this defensive check prevents further propagation of the error (see also *assert* function). In general, it is recommended that the mission-critical parts of the code, and possibly also input and output function parameters be validated. Remember, getting a fatal error during code execution is preferable to searching for elusive bugs in code that completes execution without errors.

## Advice 4: bugs and code quality

Novice programmers tend to pay little attention to how they organize the code. This is a bad practice, because when the code is messy and illegible, programmers have a higher propensity to make errors and confusion.

### Use meaningful function and variable names

Good name convention helps make sense of the logic of the code, and also serves the role of documentation. For example, when functions are named *CreateBinaryMask* or *FindPeakActivationWithinROI*, it is immediately clear what they are doing. The variables should also be given meaningful names like *voxelID* and *subjectID*, instead of *i* or *j*. The use of proper name convention for variables also decreases the chances of irrelevant data use (see Advice 1: The Dangers of Irrelevant Data). That is, the use of meaningful names makes it more difficult to get confused between variables because (a) they are less similar visually (visual saliency), and (b) they refer to specific concepts (e.g., voxels, subjects).

### Do not duplicate (“copy-paste”) your code

The main problem with this practice is that the code becomes unmanageable. That is, if the logic that was duplicated ever needs to be changed, locating and changing all of the duplicated instances is a process highly prone to error. The solution is to extract the code that needs duplication to a separate function (see next item).

### Use a modular approach

The best way to maintain and reuse the code is to adopt a modular approach. According to this method, separate business logic should be implemented in separate functions (e.g., see code samples in Box 1). The modular code is more readable because top-level flow is not cluttered by the implementation. Critically, the non-modular code contains many variables in its scope, most of which are, at any given stage of execution, completely irrelevant. This, in turn, increases the likelihood of misuse of these variables (see Advice 1: The Dangers of Irrelevant Data).

### Avoid hard-coded values

The business logic code should not contain hard-coded values unless those values are likely to never change (e.g., 60 s in minute). Initial parameters that might be changed should be either passed as parameters to the function or defined at the top of the code in a transparent way. A good practice is also to save the initial parameters for each execution, so that it would be possible to reproduce the results.

### Do not leave unsupported initial parameters (or any definitions) in the code

During code development, some initial parameters might become obsolete. For example, at the beginning, the data analysis was conducted for a single ROI that was defined as:


*INITIAL_PARAMS.ROI = 'V1.nii';*


Then, the support for multiple ROIs was added.


*INITIAL_PARAMS.ROIs_arr =…*
*   {'V1.nii', 'V2.nii', 'V3.nii'*;}


If *INITIAL_PARAMS.ROI* is no longer supported, I strongly recommend deleting or at least commenting on its definition. The main reason is that, if, by mistake, there is still a code that uses *INITIAL_PARAMS.ROI* (see Advice 1: The Dangers of Irrelevant Data), then the results of code execution would likely be wrong. By removing the obsolete definition, we ensure that no occasional use is made; if it is, then the code will throw an error (see Advice 3: Protective (preventive) Programming).

### Make your code as understandable as possible

Logic of some code might be so complicated that not only others, but even the programmer himself after some time, might struggle to understand it. Following the steps suggested above will make the code more readable and understandable. In addition, adding comments, especially to the most critical or non-trivial parts of the code, might prevent future frustration.

### Adopt peer-review procedure

Peer-review is the procedure by which you show and explain the code you wrote to your colleague(s). This is an extremely helpful procedure, not only because your colleagues might give you valuable advice, but also because explaining the code to someone else makes you look at your code more critically.

## Advice 5: consult language documentation when needed

Programming modern language, such as MATLAB or Python, is easy and intuitive. However, there are some cases when intuition is not sufficient. Consider a case when MATLAB is used in fMRI Multivariate Pattern Analysis (MVPA). A widely used preprocessing practice is to standardize a time course for each voxel (e.g., Misaki et al., 2010). To achieve this, the *zscore* function is called with a 2-D matrix of “voxels × data-volumes” parameter (e.g., Reddy et al., 2010; Lee et al., 2011; Axelrod et al., 2014). Critically, without consulting the documentation, one cannot be sure along which dimension (voxels or data-volumes) the *zscore* function makes the standardization. As a result, one can end up with an absolutely wrong output result that might look absolutely correct (i.e., correct dimensions). The solution in such cases is not to trust your intuition but to consult the documentation.

## Advice 6: take responsibility for the code you use

It is common for researchers or students to use code written by someone else in their own or another lab. There are two potential caveats here. First, the person who “inherited” the code might not fully understand how the code should be used. Second, the original code may have been written for another purpose and therefore might not work correctly in the new situation. All this might lead to dangerous bugs. The only solution is to fully understand the code you are using, regardless of whether you wrote it or not.

## Advice 7: make your code public, if possible

If you are brave enough, you might want to publish the custom code you used for your publication (e.g., Hermes et al., 2014). This permits other researchers not only to validate your code, but also to replicate your analyses (Brunet et al., 2014). Admittedly, publishing code is of limited use when the data it uses are not published, which is the case with most neuroimaging studies to date.

## Advice 8: use source management systems

The use of source management systems (e.g., https://github.com/) makes the management of code versions easy. Version control systems permit researchers to document their changes and compare between versions; critically, they permits a researcher to establish exactly when a certain change (or bug) was introduced in the code.

## Concluding remarks

This paper provided practical advice on minimizing bugs in cognitive neuroscience data analysis programming (see, summary in Table 1). Probably the most important take-home message is not to assume that the code you write is bug-free; that is, even a small piece of relatively simple code may contain a bug that could lead to incorrect results. Therefore, it is important not to be complacent and to take steps that can prevent such failures.

**Table 1:** **Summary of actions that can be taken to improve the code**.

**Type of advice**	**Actions to take**
Test your code	Use debugger to test your code	Keep track and validate intermediate results (e.g., save log file, image figures)	Validate the output of custom code using the manually created results	Check your code with simulated (surrogate) data
	Protective (preventive) programming	Make your code to check the intermediate output results as it runs
	Irrelevant data and code quality	Make sure the variables do not contain data from previous operations (e.g., loop cycle)	Extract specialized business logic to separate functions (modular approach)	Do not duplicate code	Use meaningful names for functions and variables	Avoid hard-coded values within the code	Do not leave unsupported variables (e.g., initial parameters) in the code	Add comments to the most critical and non-trivial parts of the code	Adopt peer-review procedure
		Consult documentation	Do not trust your intuition. Make sure that the functions are called according to documentation
Take responsibility for the code you use		Do not use the code without understanding it
Publish your code, if possible		Make your code available to other researchers
Use source management systems		Source systems permit to manage and document your code easily

## Conflict of interest statement

The author declares that the research was conducted in the absence of any commercial or financial relationships that could be construed as a potential conflict of interest.
